# *catena*-Poly[[(1,10-phenanthroline)zinc]-μ-2,2′-oxydibenzoato]

**DOI:** 10.1107/S160053681102856X

**Published:** 2011-07-30

**Authors:** Xue Cai

**Affiliations:** a Department of Chemistry, Mudanjiang Normal College, Mudanjiang 157012, Heilongjiang Province, People’s Republic of China

## Abstract

In the title one-dimensional coordination polymer, [Zn(C_14_H_8_O_5_)(C_12_H_8_N_2_)]_*n*_, the Zn^II^ ion is in a distorted octa­hedral coordination geometry with four O atoms from two carboxyl­ate groups in bidentate chelating modes and two N atoms from a 1,10-phenanthroline ligand. The two terminal carboxyl­ate groups bind the Zn^II^ ions, leading to a chain along the *c* axis. Adjacent chains are further linked by inter­molecular π–π inter­actions with a shortest centroid–centroid distance of 3.586 (3) Å, forming a two-dimensional supra­molecular architecture with (6,3)-network topology.

## Related literature

For related structures and the properties of coordination polymers, see, for example: Evans *et al.* (1999 ▶); Yaghi *et al.* (1998 ▶); Wang *et al.* (2005 ▶); Li *et al.* (2003 ▶). For the synthesis of 3-(4-carb­oxy­phen­oxy)phthalic acid, see: Wang *et al.* (2009 ▶).
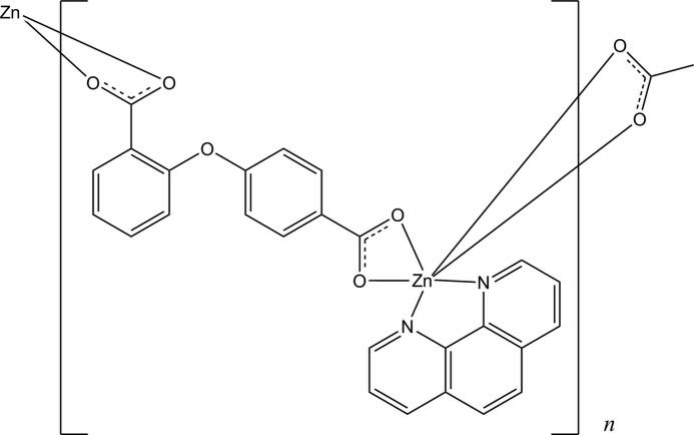


## Experimental

### 

#### Crystal data

[Zn(C_14_H_8_O_5_)(C_12_H_8_N_2_)]
*M* *_r_* = 501.78
Monoclinic, 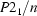

*a* = 7.7033 (18) Å
*b* = 17.403 (4) Å
*c* = 16.230 (4) Å
β = 90.184 (4)°
*V* = 2175.8 (9) Å^3^
*Z* = 4
Mo *K*α radiation
μ = 1.17 mm^−1^
*T* = 293 K
0.15 × 0.08 × 0.06 mm

#### Data collection

Bruker APEXII CCD area-detector diffractometer
Absorption correction: multi-scan (*SADABS*; Sheldrick, 2003 ▶) *T*_min_ = 0.901, *T*_max_ = 0.913
10511 measured reflections
3843 independent reflections
2320 reflections with *I* > 2σ(*I*)
*R*_int_ = 0.056

#### Refinement

*R*[*F* ^2^ > 2σ(*F* ^2^)] = 0.044
*wR*(*F* ^2^) = 0.112
*S* = 0.94
3843 reflections
307 parameters
H-atom parameters constrained
Δρ_max_ = 0.49 e Å^−3^
Δρ_min_ = −0.41 e Å^−3^

### 

Data collection: *APEX2* (Bruker, 2004 ▶); cell refinement: *SAINT-Plus* (Bruker, 2001 ▶); data reduction: *SAINT-Plus*; program(s) used to solve structure: *SHELXS97* (Sheldrick, 2008 ▶); program(s) used to refine structure: *SHELXL97* (Sheldrick, 2008 ▶); molecular graphics: *XP* in *SHELXTL* (Sheldrick, 2008 ▶); software used to prepare material for publication: *SHELXL97*.

## Supplementary Material

Crystal structure: contains datablock(s) I, global. DOI: 10.1107/S160053681102856X/is2747sup1.cif

Structure factors: contains datablock(s) I. DOI: 10.1107/S160053681102856X/is2747Isup2.hkl

Additional supplementary materials:  crystallographic information; 3D view; checkCIF report

## Figures and Tables

**Table 1:** Selected bond lengths (Å)

Zn1—O2	2.011 (2)
Zn1—N1	2.114 (3)
Zn1—N2	2.129 (3)
Zn1—O4^i^	2.143 (3)
Zn1—O5^i^	2.172 (3)
Zn1—O1	2.395 (3)

Symmetry code: (i) 
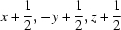
.

**Fig. 1.**
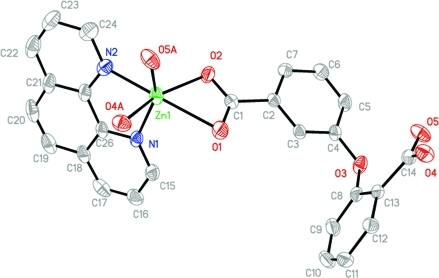
The molecular structure of the title compound. Displacement ellipsoids are drawn at 30% probability level. All hydrogen atoms have been omitted. The suffix A corresponds to symmetry code (i) in Table 1.

**Fig. 2.**
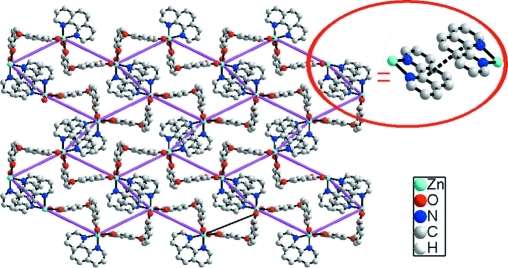
A view of the two-dimensional supramolecular architecture of the title compound.

## supplementary crystallographic information

### Comment

The design and synthesis of coordination polymers in supramolecular chemistry and crystal engineering, have been emerging as an ongoing field owing to their structural aesthetics and topologies as well as diverse functional properties (Evans *et al.*, 1999; Yaghi *et al.*, 1998) The semirigid V-shaped multicarboxylate ligands with two benzene rings bridged by an oxygen atom as central molecular framework are of increasing flexibility and therefore able to lead to metal complexes with diverse structures because of the free rotation of two benzene rings around the bridged atom (Wang *et al.*, 2005).

The compound (I) crystallizes in the monoclinic system. As shown in Fig. 1, the Zn(II) ion is located in a distorted octahedral coordination geometry completed by four oxygen atoms from two carboxyl substituents of organic carboxylic acid in a bidentate chelating mode and two nitrogen atoms from the 1,10-phenanthroline ligand. The head and terminal carboxylate groups bind Zn(II) ions to lead to a one-dimensional chain. The neighboring chains are further linked by an intermolecular π–π interaction between the phenanthroline ring systems with a shortest centroid-centroid distance 3.586 (3) Å, forming a two-dimensional supramolecular architecture (Fig. 2) with 3-connect (6,3) network topology (Li *et al.*, 2003)

### Experimental

The mixture of Zn(OAc)_2_.2H_2_O (0.044 g, 0.2 mmol), 1,10-phenanthroline (0.0360 g, 0.2 mmol), 3-(4-carboxyphenoxy)phthalic acid (H_3_L, 0.0302 g, 0.1 mmol), KOH (0.0168 g, 0.3 mmol) and H_2_O (15 ml) was sealed in 25 ml Teflon-lined stainless steel reactor, which was heated to 160 °C. Colourless block-shaped crystals suitable for X-ray diffraction analysis were separated by filtration with the yield of 0.022 g.

### Refinement

All H atoms were refined using a riding model, with C—H = 0.93 Å, and with *U*_iso_(H) = 1.2*U*_eq_(C).

### Crystal data

**Table:**

[Zn(C_14_H_8_O_5_)(C_12_H_8_N_2_)]	*F*(000) = 1024
*M**_r_* = 501.78	*D*_x_ = 1.532 Mg m^−^^3^
Monoclinic, *P*2_1_/*n*	Mo *K*α radiation, λ = 0.71073 Å
Hall symbol: -P 2yn	Cell parameters from 1776 reflections
*a* = 7.7033 (18) Å	θ = 2.3–21.9°
*b* = 17.403 (4) Å	µ = 1.17 mm^−^^1^
*c* = 16.230 (4) Å	*T* = 293 K
β = 90.184 (4)°	Block, colourless
*V* = 2175.8 (9) Å^3^	0.15 × 0.08 × 0.06 mm
*Z* = 4	

### Data collection

**Table:**

Bruker APEXII CCD area-detector diffractometer	3843 independent reflections
Radiation source: fine-focus sealed tube	2320 reflections with *I* > 2σ(*I*)
graphite	*R*_int_ = 0.056
Detector resolution: 0 pixels mm^-1^	θ_max_ = 25.0°, θ_min_ = 1.7°
φ and ω scans	*h* = −9→9
Absorption correction: multi-scan (*SADABS*; Sheldrick, 2003)	*k* = −20→16
*T*_min_ = 0.901, *T*_max_ = 0.913	*l* = −18→19
10511 measured reflections	

### Refinement

**Table:**

Refinement on *F*^2^	Primary atom site location: structure-invariant direct methods
Least-squares matrix: full	Secondary atom site location: difference Fourier map
*R*[*F*^2^ > 2σ(*F*^2^)] = 0.044	Hydrogen site location: inferred from neighbouring sites
*wR*(*F*^2^) = 0.112	H-atom parameters constrained
*S* = 0.94	*w* = 1/[σ^2^(*F*_o_^2^) + (0.0558*P*)^2^] where *P* = (*F*_o_^2^ + 2*F*_c_^2^)/3
3843 reflections	(Δ/σ)_max_ = 0.001
307 parameters	Δρ_max_ = 0.49 e Å^−^^3^
0 restraints	Δρ_min_ = −0.41 e Å^−^^3^

### Special details

**Table:**

Geometry. All e.s.d.'s (except the e.s.d. in the dihedral angle between two l.s. planes) are estimated using the full covariance matrix. The cell e.s.d.'s are taken into account individually in the estimation of e.s.d.'s in distances, angles and torsion angles; correlations between e.s.d.'s in cell parameters are only used when they are defined by crystal symmetry. An approximate (isotropic) treatment of cell e.s.d.'s is used for estimating e.s.d.'s involving l.s. planes.
Refinement. Refinement of *F*^2^ against ALL reflections. The weighted *R*-factor *wR* and goodness of fit *S* are based on *F*^2^, conventional *R*-factors *R* are based on *F*, with *F* set to zero for negative *F*^2^. The threshold expression of *F*^2^ > σ(*F*^2^) is used only for calculating *R*-factors(gt) *etc*. and is not relevant to the choice of reflections for refinement. *R*-factors based on *F*^2^ are statistically about twice as large as those based on *F*, and *R*- factors based on ALL data will be even larger.

### Fractional atomic coordinates and isotropic or equivalent isotropic displacement parameters (Å^2^)

**Table:**

	*x*	*y*	*z*	*U*_iso_*/*U*_eq_	
Zn1	0.13817 (6)	0.13631 (3)	0.83696 (3)	0.04575 (18)	
O3	−0.2343 (3)	0.10169 (15)	0.42299 (15)	0.0501 (7)	
O4	−0.1006 (3)	0.32154 (16)	0.33955 (16)	0.0508 (7)	
N1	0.2187 (4)	0.02020 (18)	0.83600 (18)	0.0427 (8)	
O5	−0.3157 (3)	0.24285 (15)	0.36218 (17)	0.0576 (8)	
O2	−0.0997 (3)	0.14036 (15)	0.78576 (15)	0.0505 (7)	
C25	0.2064 (5)	0.0400 (2)	0.9814 (2)	0.0449 (10)	
O1	0.1008 (3)	0.14258 (19)	0.69057 (16)	0.0687 (9)	
N2	0.1358 (4)	0.1102 (2)	0.96511 (19)	0.0488 (8)	
C18	0.3184 (5)	−0.0821 (3)	0.9262 (3)	0.0527 (11)	
C26	0.2485 (4)	−0.0083 (2)	0.9126 (2)	0.0431 (10)	
C1	−0.0532 (5)	0.1416 (2)	0.7110 (2)	0.0446 (10)	
C10	0.2189 (6)	0.0738 (3)	0.3682 (3)	0.0603 (12)	
H10	0.2996	0.0340	0.3675	0.072*	
C13	−0.0274 (4)	0.1917 (2)	0.3654 (2)	0.0366 (9)	
C11	0.2659 (5)	0.1464 (3)	0.3426 (2)	0.0568 (12)	
H11	0.3793	0.1561	0.3262	0.068*	
C5	−0.4461 (5)	0.1157 (2)	0.5263 (3)	0.0558 (11)	
H5	−0.5305	0.1067	0.4864	0.067*	
C21	0.2411 (5)	0.0138 (3)	1.0612 (3)	0.0599 (13)	
C12	0.1449 (4)	0.2045 (2)	0.3413 (2)	0.0431 (9)	
H12	0.1781	0.2533	0.3242	0.052*	
C3	−0.1465 (4)	0.1308 (2)	0.5634 (2)	0.0379 (9)	
H3	−0.0304	0.1345	0.5483	0.046*	
C14	−0.1562 (5)	0.2555 (2)	0.3559 (2)	0.0403 (9)	
C2	−0.1942 (4)	0.1381 (2)	0.6454 (2)	0.0369 (8)	
C4	−0.2722 (5)	0.1181 (2)	0.5043 (2)	0.0407 (9)	
C15	0.2555 (5)	−0.0236 (3)	0.7719 (3)	0.0528 (11)	
H15	0.2365	−0.0042	0.7193	0.063*	
C9	0.0534 (6)	0.0604 (2)	0.3947 (2)	0.0559 (11)	
H9	0.0222	0.0116	0.4127	0.067*	
C17	0.3525 (5)	−0.1265 (3)	0.8556 (3)	0.0688 (13)	
H17	0.3966	−0.1760	0.8613	0.083*	
C22	0.2001 (6)	0.0634 (4)	1.1255 (3)	0.0769 (16)	
H22	0.2193	0.0482	1.1797	0.092*	
C8	−0.0682 (5)	0.1189 (2)	0.3951 (2)	0.0400 (9)	
C16	0.3218 (5)	−0.0979 (3)	0.7799 (3)	0.0649 (13)	
H16	0.3446	−0.1274	0.7334	0.078*	
C20	0.3170 (6)	−0.0615 (3)	1.0716 (3)	0.0721 (15)	
H20	0.3415	−0.0793	1.1244	0.086*	
C24	0.0998 (6)	0.1566 (3)	1.0270 (3)	0.0647 (13)	
H24	0.0525	0.2048	1.0164	0.078*	
C6	−0.4932 (5)	0.1267 (3)	0.6066 (3)	0.0585 (12)	
H6	−0.6100	0.1275	0.6208	0.070*	
C23	0.1324 (7)	0.1338 (4)	1.1098 (3)	0.0764 (15)	
H23	0.1073	0.1671	1.1530	0.092*	
C7	−0.3675 (5)	0.1366 (2)	0.6669 (2)	0.0484 (10)	
H7	−0.3998	0.1422	0.7218	0.058*	
C19	0.3527 (6)	−0.1059 (3)	1.0072 (3)	0.0713 (14)	
H19	0.4017	−0.1541	1.0161	0.086*	

### Atomic displacement parameters (Å^2^)

**Table:**

	*U*^11^	*U*^22^	*U*^33^	*U*^12^	*U*^13^	*U*^23^
Zn1	0.0362 (3)	0.0535 (3)	0.0475 (3)	−0.0081 (2)	−0.0086 (2)	0.0039 (2)
O3	0.0465 (17)	0.0655 (19)	0.0382 (15)	−0.0152 (14)	−0.0071 (13)	0.0035 (13)
O4	0.0418 (16)	0.0465 (17)	0.0641 (18)	0.0019 (13)	0.0034 (13)	0.0047 (14)
N1	0.0335 (17)	0.056 (2)	0.0385 (19)	−0.0111 (15)	−0.0066 (14)	0.0001 (17)
O5	0.0310 (16)	0.0534 (18)	0.088 (2)	0.0039 (13)	−0.0074 (14)	−0.0064 (15)
O2	0.0432 (16)	0.074 (2)	0.0344 (15)	−0.0024 (14)	−0.0046 (12)	−0.0032 (14)
C25	0.031 (2)	0.060 (3)	0.044 (2)	−0.0180 (19)	−0.0070 (18)	0.005 (2)
O1	0.0240 (15)	0.129 (3)	0.0530 (17)	−0.0026 (16)	−0.0029 (13)	−0.0030 (18)
N2	0.042 (2)	0.060 (2)	0.045 (2)	−0.0138 (17)	0.0019 (16)	−0.0062 (18)
C18	0.033 (2)	0.065 (3)	0.060 (3)	−0.008 (2)	−0.009 (2)	0.011 (2)
C26	0.027 (2)	0.057 (3)	0.046 (2)	−0.0136 (18)	−0.0087 (17)	0.007 (2)
C1	0.038 (2)	0.052 (3)	0.044 (2)	0.0057 (19)	−0.0039 (19)	−0.002 (2)
C10	0.055 (3)	0.061 (3)	0.065 (3)	0.025 (2)	−0.017 (2)	−0.012 (3)
C13	0.033 (2)	0.046 (2)	0.030 (2)	0.0025 (17)	−0.0037 (16)	−0.0015 (17)
C11	0.037 (2)	0.084 (4)	0.049 (3)	0.013 (2)	−0.0012 (19)	−0.010 (2)
C5	0.035 (2)	0.081 (3)	0.051 (3)	−0.003 (2)	−0.015 (2)	0.003 (2)
C21	0.037 (2)	0.102 (4)	0.040 (3)	−0.025 (2)	−0.006 (2)	0.009 (3)
C12	0.032 (2)	0.052 (2)	0.045 (2)	−0.002 (2)	0.0015 (17)	0.004 (2)
C3	0.0273 (19)	0.047 (2)	0.039 (2)	0.0002 (17)	−0.0036 (16)	0.0049 (18)
C14	0.035 (2)	0.049 (3)	0.036 (2)	0.0019 (19)	−0.0065 (17)	−0.0057 (18)
C2	0.0287 (19)	0.042 (2)	0.040 (2)	0.0005 (17)	−0.0034 (16)	0.0002 (18)
C4	0.036 (2)	0.047 (2)	0.039 (2)	−0.0042 (17)	−0.0059 (17)	0.0071 (18)
C15	0.040 (2)	0.069 (3)	0.049 (3)	−0.006 (2)	−0.0082 (19)	−0.005 (2)
C9	0.064 (3)	0.051 (3)	0.053 (3)	0.012 (2)	−0.014 (2)	0.001 (2)
C17	0.040 (3)	0.059 (3)	0.107 (4)	0.004 (2)	−0.008 (3)	0.005 (3)
C22	0.058 (3)	0.133 (5)	0.040 (3)	−0.032 (3)	−0.002 (2)	0.007 (3)
C8	0.039 (2)	0.051 (3)	0.030 (2)	−0.0003 (19)	−0.0073 (17)	−0.0003 (18)
C16	0.040 (3)	0.074 (3)	0.081 (3)	0.002 (2)	−0.010 (2)	−0.020 (3)
C20	0.043 (3)	0.111 (5)	0.062 (3)	−0.016 (3)	−0.014 (2)	0.047 (3)
C24	0.053 (3)	0.081 (4)	0.060 (3)	−0.017 (2)	0.008 (2)	−0.012 (3)
C6	0.025 (2)	0.089 (3)	0.062 (3)	−0.001 (2)	−0.004 (2)	−0.002 (3)
C23	0.064 (3)	0.120 (5)	0.045 (3)	−0.028 (3)	0.007 (2)	−0.024 (3)
C7	0.038 (2)	0.063 (3)	0.044 (2)	0.005 (2)	0.0032 (18)	0.001 (2)
C19	0.043 (3)	0.091 (4)	0.080 (4)	−0.005 (3)	−0.009 (3)	0.032 (3)

### Geometric parameters (Å, °)

**Table:**

Zn1—O2	2.011 (2)	C11—H11	0.9300
Zn1—N1	2.114 (3)	C5—C6	1.369 (6)
Zn1—N2	2.129 (3)	C5—C4	1.387 (5)
Zn1—O4^i^	2.143 (3)	C5—H5	0.9300
Zn1—O5^i^	2.172 (3)	C21—C22	1.391 (7)
Zn1—O1	2.395 (3)	C21—C20	1.446 (7)
O3—C4	1.383 (4)	C12—H12	0.9300
O3—C8	1.392 (4)	C3—C4	1.378 (5)
O4—C14	1.255 (4)	C3—C2	1.389 (5)
N1—C15	1.321 (5)	C3—H3	0.9300
N1—C26	1.358 (4)	C2—C7	1.381 (5)
O5—C14	1.253 (4)	C15—C16	1.396 (6)
O2—C1	1.266 (4)	C15—H15	0.9300
C25—N2	1.362 (5)	C9—C8	1.383 (5)
C25—C21	1.398 (5)	C9—H9	0.9300
C25—C26	1.437 (5)	C17—C16	1.345 (6)
O1—C1	1.233 (4)	C17—H17	0.9300
N2—C24	1.319 (5)	C22—C23	1.355 (7)
C18—C19	1.402 (6)	C22—H22	0.9300
C18—C17	1.407 (6)	C16—H16	0.9300
C18—C26	1.410 (6)	C20—C19	1.329 (6)
C1—C2	1.520 (5)	C20—H20	0.9300
C10—C9	1.367 (6)	C24—C23	1.423 (6)
C10—C11	1.379 (6)	C24—H24	0.9300
C10—H10	0.9300	C6—C7	1.385 (5)
C13—C8	1.393 (5)	C6—H6	0.9300
C13—C12	1.403 (5)	C23—H23	0.9300
C13—C14	1.496 (5)	C7—H7	0.9300
C11—C12	1.375 (5)	C19—H19	0.9300
			
O2—Zn1—N1	107.31 (11)	C25—C21—C20	118.6 (4)
O2—Zn1—N2	113.60 (11)	C11—C12—C13	121.4 (4)
N1—Zn1—N2	78.85 (13)	C11—C12—H12	119.3
O2—Zn1—O4^i^	148.31 (11)	C13—C12—H12	119.3
N1—Zn1—O4^i^	92.97 (11)	C4—C3—C2	119.6 (3)
N2—Zn1—O4^i^	93.73 (11)	C4—C3—H3	120.2
O2—Zn1—O5^i^	101.10 (10)	C2—C3—H3	120.2
N1—Zn1—O5^i^	151.55 (10)	O5—C14—O4	120.9 (3)
N2—Zn1—O5^i^	91.41 (12)	O5—C14—C13	120.8 (4)
O4^i^—Zn1—O5^i^	60.75 (10)	O4—C14—C13	118.3 (3)
O2—Zn1—O1	58.81 (10)	O5—C14—Zn1^ii^	61.1 (2)
N1—Zn1—O1	94.05 (11)	O4—C14—Zn1^ii^	59.79 (19)
N2—Zn1—O1	167.82 (11)	C13—C14—Zn1^ii^	177.9 (3)
O4^i^—Zn1—O1	96.52 (10)	C7—C2—C3	119.9 (3)
O5^i^—Zn1—O1	99.32 (11)	C7—C2—C1	120.9 (3)
C4—O3—C8	117.7 (3)	C3—C2—C1	119.0 (3)
C14—O4—Zn1^ii^	89.8 (2)	C3—C4—O3	123.2 (3)
C15—N1—C26	118.3 (4)	C3—C4—C5	120.2 (4)
C15—N1—Zn1	128.4 (3)	O3—C4—C5	116.5 (3)
C26—N1—Zn1	113.1 (3)	N1—C15—C16	122.7 (4)
C14—O5—Zn1^ii^	88.5 (2)	N1—C15—H15	118.6
C1—O2—Zn1	97.8 (2)	C16—C15—H15	118.6
N2—C25—C21	123.2 (4)	C10—C9—C8	120.6 (4)
N2—C25—C26	117.7 (3)	C10—C9—H9	119.7
C21—C25—C26	119.1 (4)	C8—C9—H9	119.7
C1—O1—Zn1	81.1 (2)	C16—C17—C18	120.5 (4)
C24—N2—C25	119.0 (4)	C16—C17—H17	119.8
C24—N2—Zn1	128.0 (3)	C18—C17—H17	119.8
C25—N2—Zn1	112.1 (3)	C23—C22—C21	120.5 (5)
C19—C18—C17	124.5 (5)	C23—C22—H22	119.7
C19—C18—C26	119.1 (4)	C21—C22—H22	119.7
C17—C18—C26	116.5 (4)	C9—C8—O3	117.8 (4)
N1—C26—C18	122.7 (4)	C9—C8—C13	121.0 (4)
N1—C26—C25	117.4 (4)	O3—C8—C13	121.1 (3)
C18—C26—C25	120.0 (4)	C17—C16—C15	119.4 (5)
O1—C1—O2	122.3 (3)	C17—C16—H16	120.3
O1—C1—C2	119.9 (3)	C15—C16—H16	120.3
O2—C1—C2	117.8 (3)	C19—C20—C21	121.4 (4)
O1—C1—Zn1	70.0 (2)	C19—C20—H20	119.3
O2—C1—Zn1	52.30 (17)	C21—C20—H20	119.3
C2—C1—Zn1	169.3 (3)	N2—C24—C23	120.8 (5)
C9—C10—C11	119.8 (4)	N2—C24—H24	119.6
C9—C10—H10	120.1	C23—C24—H24	119.6
C11—C10—H10	120.1	C5—C6—C7	120.3 (4)
C8—C13—C12	117.1 (3)	C5—C6—H6	119.9
C8—C13—C14	124.1 (3)	C7—C6—H6	119.9
C12—C13—C14	118.8 (3)	C22—C23—C24	119.8 (5)
C12—C11—C10	120.0 (4)	C22—C23—H23	120.1
C12—C11—H11	120.0	C24—C23—H23	120.1
C10—C11—H11	120.0	C2—C7—C6	119.9 (4)
C6—C5—C4	120.0 (4)	C2—C7—H7	120.1
C6—C5—H5	120.0	C6—C7—H7	120.1
C4—C5—H5	120.0	C20—C19—C18	121.8 (5)
C22—C21—C25	116.7 (5)	C20—C19—H19	119.1
C22—C21—C20	124.7 (5)	C18—C19—H19	119.1

Symmetry codes: (i) *x*+1/2, −*y*+1/2, *z*+1/2; (ii) *x*−1/2, −*y*+1/2, *z*−1/2.
